# Factors Associated with Malnutrition Among Bedridden Older Adults in Nakhon Si Thammarat, Thailand: A Secondary Cross-Sectional Analysis

**DOI:** 10.3390/medsci14060606

**Published:** 2026-09-26

**Authors:** Sopida Thipsawat, Rachadaporn Jantasuwan, Tipwan Ponthophong, Ruth B. Grubesic

**Affiliations:** 1 School of Nursing, The Excellence Center of Community Health Promotion, Walailak University, Nakhon Si Thammarat 80161, Thailand; s.thipsawat@gmail.com; 2 Pak Phaya Subdistrict Health Promoting Hospital, Nakhon Si Thammarat 80000, Thailand; nandhachai07@gmail.com; 3 Nursing and Public Health Sciences, Schreiner University Main Campus, 2100 Memorial Blvd., Kerrville, TX 78028, USA; rbgrubesic@schreiner.edu

**Keywords:** bedridden older adults, malnutrition, nutritional status, mini nutritional assessment, waist circumference

## Abstract

Background/Objectives: Malnutrition is a major concern among bedridden older adults because functional limitations and dependence on others may compromise adequate nutritional intake. This study aimed to identify factors associated with malnutrition among bedridden older adults in Nakhon Si Thammarat Province, Thailand. Methods: This cross-sectional analytical study used secondary data from 210 bedridden older adults. Nutritional status was assessed using the 18-item Mini Nutritional Assessment (MNA^®^). Because only two participants had normal nutritional status, they were excluded from the regression analysis due to the insufficient number of observations in this category. The analytical sample therefore comprised 208 participants, and nutritional status was analyzed as a binary outcome: at risk of malnutrition or malnutrition. Demographic, functional, anthropometric, oral health, and dietary factors were examined. Group differences were assessed using the chi-square test or Fisher’s exact test for categorical variables and the Mann–Whitney U test for continuous variables. Multivariable binary logistic regression was used to identify factors independently associated with malnutrition. Results: Among the 208 participants included in the analysis, 40 (19.23%) were at risk of malnutrition and 168 (80.77%) were malnourished. These proportions refer to the analytical sample after exclusion of the two participants with normal nutritional status and do not represent the prevalence of malnutrition in the original sample. In the multivariable analysis, female sex was associated with higher odds of malnutrition (AOR = 2.302, 95% CI: 1.011–5.238, *p* = 0.047), while greater waist circumference was associated with lower odds of malnutrition (AOR = 0.948, 95% CI: 0.921–0.976, *p* < 0.001). Conclusions: Sex and waist circumference were independently associated with malnutrition among bedridden older adults with compromised nutritional status. Given the cross-sectional design, these associations should not be interpreted as causal or protective. The findings support nutritional assessment and anthropometric monitoring as part of caregiver-supported care.

## 1. Introduction

The global population is aging rapidly, with the number of older adults expected to increase substantially in the coming decades. The population aged 60 years and older is projected to double from approximately 1 billion in 2020 to 2.1 billion by 2050. Moreover, nearly 80% of older adults are expected to live in low- and middle-income countries by 2050 [1]. This demographic transition is also evident in Thailand, which officially became a fully aged society in 2021, when individuals aged 60 years and older accounted for approximately 20% of the total population, or around 13 million people. By 2040, Thailand’s older population is projected to reach 20 million, accounting for approximately one-third of the country’s total population [2].

The aging process is often accompanied by physiological and sensory changes that may adversely affect appetite [3] and thirst regulation [4]. Anorexia is estimated to affect approximately 20–30% of older adults [5]. Age-related reductions in gastric acid secretion may interfere with the absorption of essential nutrients, particularly iron and vitamin B12. Reduced salivary secretion and other gastrointestinal changes may also contribute to impaired digestive function and constipation [6]. In addition, chewing and swallowing difficulties, acute and chronic illnesses, and complex medication regimens may negatively affect nutritional status among older adults [3]. Oral health, particularly the number of remaining natural teeth, may also play an important role in maintaining adequate nutritional status. Recent evidence from older nursing home residents showed that individuals with at least 20 natural teeth had substantially higher odds of having normal nutritional status than those without natural teeth, even after adjustment for potential confounding factors (OR = 4.29, 95% CI: 2.26–6.15) [7]. Similarly, the presence of natural tooth pairs in the posterior region has been associated with a lower prevalence of nutritional risk. Each additional pair of natural premolars and molars was associated with a 15.40% and 22.80% reduction in the prevalence ratio (PR) of nutritional risk, respectively (*p* < 0.05) [8].

Malnutrition, defined as deficiencies, excesses, or imbalances in energy and/or nutrient intake [9], is an important health concern in older adults. It may manifest as unintended weight loss, low body mass index (BMI), or micronutrient deficiencies [10], with age-related frailty and declining physiological reserves further increasing vulnerability [11]. Malnutrition is associated with impaired physiological function [12], infections, sarcopenia and mortality [13], reduced physical function and frailty [14], poorer quality of life [15], and increased healthcare costs [16]. Previous research has identified multiple factors associated with nutritional status among older adults, which can be broadly categorized into sociodemographic, anthropometric, functional, and oral health-related factors. Regarding sociodemographic factors, a study involving 616 older adults from both urban and rural areas found that sex was significantly associated with malnutrition among rural residents (AOR = 2.2, 95% CI: 1.0–4.8) [17]. Consistent with this finding, a community-based cross-sectional study of 739 randomly selected adults aged over 60 years in eastern Ethiopia found that female sex was independently associated with higher odds of malnutrition (AOR 1.6; 95% CI 1.1–2.2) [15]. Furthermore, this finding is consistent with a previous systematic review and meta-analysis of risk factors for malnutrition among older Thai adults, which identified female sex as a factor associated with malnutrition [18].

Anthropometric characteristics have also been linked to nutritional status in older adults. A study involving 182 community-dwelling adults aged 65 years and older found that lower waist circumference was independently associated with an increased risk of malnutrition (OR = 1.089, 95% CI: 1.040–1.140, *p* < 0.001) [19]. In addition, physical functional status, particularly dependence in activities of daily living, has consistently emerged as an important factor associated with malnutrition. Among 2323 participants aged ≥ 60 years, multivariable binary logistic regression analysis identified complete physical functional dependence as the strongest independent risk factor for malnutrition (OR 4.46, 95% CI 2.92, 6.82), with physical function assessed using the Barthel Index (BI) [20]. Similarly, a study involving 365 patients found that dependence in activities of daily living, as assessed using the Barthel ADL Index, was independently associated with malnutrition (OR = 3.81, 95% CI: 2.61–5.57) [21]. Oral health-related factors may also contribute to nutritional status in older adults. A study involving 986 older adults aged ≥65 years recruited from 29 long-term care insurance facilities across 15 regions of Japan found that BMI <20 kg/m^2^ was significantly associated with the number of molar occlusions (OR = 0.52–0.70, 95% CI: 0.28–1.00) [22]. Collectively, these findings suggest that malnutrition in older adults is influenced by multiple interrelated factors, encompassing sociodemographic characteristics, anthropometric status, physical functional dependence, and oral health.

In Thailand, the growing older population is increasingly affected by age-related physical decline, chronic diseases [23], and disabilities that may eventually result in bedridden status [24]. Bedridden older adults may be particularly susceptible to malnutrition, as functional impairment, limited mobility, difficulties with eating, and dependence on caregivers may interfere with adequate dietary intake [20,21]. In addition, this population may experience distinct nutritional vulnerabilities associated with a high level of dependence in activities of daily living, changes in body composition, oral health limitations, and reliance on caregivers for food intake. However, evidence regarding the roles of these factors and their associations with malnutrition among bedridden older adults remains limited, particularly in southern Thailand.

## 2. Materials and Methods

### 2.1. Study Design and Population

This study employed a cross-sectional analytical design using secondary data derived from a portion of the research project entitled “The Relationship Between Nutrient Consumption, Food Types, and Nutritional Status in Elderly Bedridden in Nakhon Si Thammarat Province,” with permission from the principal investigator. The original study was conducted in primary care units across Nakhon Si Thammarat Province, Thailand, between February and June 2021.

In the original study, the source population comprised 1604 bedridden older adults residing in Nakhon Si Thammarat Province, Thailand, as of 30 September 2020. The sample size for the original study was determined using G*Power version 3.1.9.4 based on a χ^2^ goodness-of-fit test for contingency tables, assuming an effect size of 0.30, a significance level (α) of 0.05, a statistical power of 0.95, and 3 degrees of freedom. The analysis indicated a minimum required sample size of 191 participants. To account for a potential 10% loss or incomplete data, the target sample size was increased to 210 participants. A multistage sampling approach was used in the original study. First, purposive sampling was used to select four districts with the largest numbers of bedridden older adults in Nakhon Si Thammarat Province. Second, quota sampling was used to allocate the required sample proportionally according to the number of bedridden older adults in each selected district. Finally, simple random sampling was used to select eligible bedridden older adults from primary care units within each district according to the calculated allocation. The present study used secondary data from these 210 participants.

Participants were eligible if they met the following inclusion criteria: (1) had functional impairment and dependence in activities of daily living, defined as a Barthel Activities of Daily Living (ADL) Index score of 0–4; (2) the bedridden older adult and/or caregiver provided consent to participate and provide the required information; (3) a primary caregiver was available to provide complete information when the older adult was unable to communicate; and (4) the older adult had been bedridden for at least 40 days. Participants were excluded if (1) the bedridden older adult and/or primary caregiver declined to provide information during data collection or (2) the older adult had an acute or critical illness requiring hospitalization. Of the 210 participants, only two were classified as having normal nutritional status according to the Mini Nutritional Assessment (MNA^®^). Because this category contained too few participants for meaningful comparison and multivariable analysis, these two participants were excluded from the analytical sample. Consequently, the final analysis included 208 participants, and nutritional status was analyzed as a binary outcome comprising at risk of malnutrition and malnutrition.

### 2.2. Data Collection Procedure

For the present study, the researchers conducted a secondary analysis of a subset of data obtained from the research project entitled “The Relationship Between Nutrient Consumption, Food Types, and Nutritional Status in Elderly Bedridden in Nakhon Si Thammarat Province,” with permission from the principal investigator.

### 2.3. Research Instruments

Data were extracted using a structured data extraction form developed by the researchers. Variables included in the data extraction form were selected based on a review of the relevant literature, with the medical model serving as an overarching framework for identifying individual health-related factors potentially associated with malnutrition among bedridden older adults. The extracted variables were organized into four sections: (1) demographic and socioeconomic characteristics, (2) health, functional, oral health, and anthropometric characteristics, (3) dietary intake, and (4) nutritional status.

Section 1: Demographic and socioeconomic characteristics. This section included sex, age, religion, marital status, duration of bedridden status, number of family members, primary caregiver, monthly household income, and income sufficiency.

Section 2: Health, functional, oral health, and anthropometric characteristics. Health and functional characteristics included ability to eat and functional status assessed using the Barthel ADL Index. Oral health characteristics included the number of remaining teeth and number of molar teeth. Anthropometric measures included waist circumference, hip circumference, calf circumference, mid-upper-arm circumference, half-arm span, and knee height, all measured in centimeters. Waist circumference was measured at the midpoint between the lower margin of the last rib and the iliac crest. Hip circumference was measured around the widest part of the buttocks. Calf circumference was measured with the participant in a supine position and the knee flexed at 90°. A measuring tape was placed around the widest part of the left calf, ensuring that it was neither too loose nor too tight. If edema was present in the left calf, the measurement was taken from the right calf instead. Mid-upper-arm circumference was measured on the left arm at the midpoint between the acromion process and the olecranon process. If edema was present in the left arm, the measurement was taken from the right arm instead. Half-arm span was measured with the participant’s arm fully extended perpendicular to the trunk, from the tip of the middle finger to the midpoint of the suprasternal notch. Knee height was measured from the heel to the knee and recorded in centimeters.

Because the participants were bedridden older adults for whom direct measurement of body weight and standing height was difficult and appropriate weighing and height-measuring equipment was not readily applicable, anthropometric measurements were used to estimate body weight and height. Each anthropometric measurement was performed three times to minimize measurement error, and the mean of the three measurements was used for subsequent calculations. For participants with a condition affecting one side of the body, such as unilateral edema, measurements were taken on the unaffected side. Estimated height was calculated from knee height and age using sex-specific equations [25]:

For men:

Estimated height (cm) = (2.02 × knee height [cm]) − (0.40 × age [years]) + 64.19

For women:

Estimated height (cm) = (1.83 × knee height [cm]) − (0.24 × age [years]) + 84.88

Estimated body weight was calculated using sex-specific equations incorporating waist circumference, hip circumference, calf circumference, mid-upper-arm circumference, and half-arm span [26]:

For men:

Estimated weight (kg) = −85.93 + (0.27 × waist circumference [cm]) + (0.59 × hip circumference [cm]) + (0.43 × calf circumference [cm]) + (0.89 × mid-upper-arm circumference [cm]) + (0.40 × half-arm span [cm])

For women:

Estimated weight (kg) = −76.86 + (0.24 × waist circumference [cm]) + (0.36 × hip circumference [cm]) + (0.66 × calf circumference [cm]) + (0.49 × mid-upper-arm circumference [cm]) + (0.53 × half-arm span [cm])

Body mass index (BMI) was subsequently calculated from the estimated body weight and height using the following equations:

BMI (kg/m^2^) = estimated body weight (kg)/estimated height^2^ (m^2^).

Thus, waist circumference, hip circumference, calf circumference, mid-upper-arm circumference, half-arm span, and knee height were directly measured anthropometric variables, whereas body weight, height, and BMI were derived from these measurements using the specified equations.

Section 3: Dietary intake. Dietary variables used in the present study were obtained from the dataset of the original study. These variables included carbohydrate, protein, and fat intake, expressed as kilocalories (kcal), as well as total energy intake. In the original study, dietary intake data were collected from primary caregivers of bedridden older adults using 24 h dietary recalls. Caregivers reported the foods and beverages consumed by the older adults during the preceding 24 h, including food items, meal components, and quantities consumed. Dietary information was collected for three days, comprising two weekdays and one weekend day. Energy and nutrient intakes were analyzed using INMUCAL Version 4.0 (Institute of Nutrition, Mahidol University, Nakhon Pathom, Thailand), developed by the Institute of Nutrition, Mahidol University, Thailand. The average daily intake of each nutrient was calculated from the three 24 h dietary recalls and used as the dietary intake data in the present secondary analysis.

Section 4: Nutritional status. Nutritional status, the dependent variable in this study, was assessed using the 18-item MNA^®^. The total MNA^®^ score ranges from 0 to 30 and classifies nutritional status into three categories: normal nutritional status (24–30 points), at risk of malnutrition (17–23.5 points), and malnutrition (<17 points) [27]. The MNA^®^ is a well-validated screening tool for older adults. The original validation study of the full MNA^®^ demonstrated a sensitivity of 96%, specificity of 98%, and positive predictive value of 97% when compared with clinical status. The MNA^®^ was also validated against two principal criteria, clinical status and comprehensive nutritional assessment, using principal component and discriminant analyses [28]. In addition, the MNA^®^ is a simplified assessment tool that can be used by adults aged 65 years and older or their caregivers. It has been scientifically validated and shown to be as effective as the MNA^®^ in identifying malnutrition [27].

### 2.4. Statistical Analysis

The researcher collected the study data and performed the statistical analyses using IBM SPSS Statistics, version 23. A statistical significance level of 0.05 was established. Descriptive statistics were used to summarize the demographic, health-related, anthropometric, and dietary characteristics of the participants. Categorical variables were presented as frequencies and percentages, whereas continuous variables were summarized using medians and interquartile ranges (Q1–Q3). Differences between participants at risk of malnutrition and those with malnutrition were examined using the Chi-square test or Fisher’s exact test, as appropriate, for categorical variables and the Mann–Whitney U test for continuous variables.

Variables with a *p*-value < 0.20 in the chi-square test, Fisher’s exact test, or Mann–Whitney U test, together with relevant demographic characteristics, were subsequently entered into a multivariable binary logistic regression model to identify factors independently associated with malnutrition. In addition, variables considered clinically relevant to nutritional status were considered where appropriate. Although BMI, calf circumference, and mid-upper-arm circumference yielded *p*-values < 0.20 in the bivariate analyses using the Mann–Whitney U test, Chi-square test or Fisher’s exact test, these variables were excluded from the multivariable model because they are components of the MNA^®^, which was used to assess the dependent variable. Their inclusion as independent variables could therefore result in conceptual overlap with the nutritional-status outcome.

Prior to multivariable analysis, the assumptions and diagnostic requirements for binary logistic regression were assessed. Multicollinearity among the candidate independent variables was evaluated using tolerance and variance inflation factor (VIF) values. Tolerance values ranged from 0.707 to 0.930, while VIF values ranged from 1.076 to 1.414, indicating no evidence of problematic multicollinearity. The linearity of the logit for continuous independent variables was assessed using the Box–Tidwell procedure by examining interaction terms between each continuous independent variable and its natural logarithmic transformation. For continuous variables containing zero values, a constant of 1 was added to all observations before applying the natural logarithmic transformation to avoid undefined logarithmic values [i.e., ln(X + 1)]. The corresponding interaction terms were then constructed using the transformed variables. Potential outliers and influential observations were evaluated using standardized residuals, Cook’s distance, and leverage values. Observations with relatively large diagnostic values were individually reviewed against the original dataset and retained when confirmed to represent valid and plausible observations. No missing data were identified for the variables included in the final regression model; therefore, all 208 participants were included in the multivariable analysis.

Multivariable binary logistic regression using the enter method was subsequently performed to identify factors independently associated with malnutrition. Nutritional status was specified as the binary dependent variable, with participants at risk of malnutrition coded as 0 and those with malnutrition coded as 1. The final model included sex, ability to eat, waist circumference, Barthel ADL score, number of molar teeth, carbohydrate intake, protein intake, and fat intake. Sex and ability to eat were treated as categorical variables, whereas waist circumference, Barthel ADL score, number of molar teeth, and macronutrient intakes were entered as continuous variables. Adjusted odds ratios (AORs) with 95% confidence intervals (CIs) were reported.

### 2.5. Ethical Approval

This study involved a secondary analysis of selected data from the research project entitled “The Relationship Between Nutrient Consumption, Food Types, and Nutritional Status in Elderly Bedridden in Nakhon Si Thammarat Province.” Ethical review for the present study was conducted by the Institutional Review Board of Walailak University, Thailand, which granted exemption review approval (Approval No. WUEC-24-222-02) on 25 June 2025, with the approval valid for one year. All data used in the analysis were handled in accordance with the approved ethical requirements and applicable principles for research involving human participants.

## 3. Results

A total of 210 bedridden older adults were initially included in the study. Of these, 2 (0.95%) had normal nutritional status, 40 (19.05%) were at risk of malnutrition, and 168 (80.00%) were malnourished. Because only two participants had normal nutritional status, this category was too small for meaningful comparison and multivariable analysis. These two participants were therefore excluded from the analytical sample, resulting in a final sample of 208 participants. Among those included in the analysis, 40 (19.23%) were at risk of malnutrition and 168 (80.77%) were malnourished.

### 3.1. Demographic and Health-Related Characteristics

Among the 208 participants, the median age was 82.00 years (Q1–Q3: 73.00–89.00). Most participants were female (63.46%), Buddhist (89.42%), and divorced, widowed, or separated (51.92%). The median duration of being bedridden was 30.00 months (Q1–Q3: 12.00–50.25). Regarding caregiving arrangements, children were the most common primary caregivers, accounting for 59.62% of participants. In addition, nearly two-thirds of the participants (63.94%) reported insufficient household income.

Ability to eat differed significantly between participants at risk of malnutrition and those with malnutrition (χ^2^ = 9.847, *p* = 0.020). Self-feeding was more common among participants at risk of malnutrition than among those with malnutrition (40.00% vs. 19.64%), whereas being fed by a caregiver (35.00% vs. 45.83%) and tube feeding (2.50% vs. 13.10%) were more common among participants with malnutrition. The proportion of participants who were both self-feeding and fed by a caregiver was similar between the two groups (22.50% vs. 21.43%, respectively). No statistically significant differences between the two groups were observed for sex, age, religion, marital status, duration of being bedridden, number of family members, primary caregiver, monthly household income, or income sufficiency (all *p* > 0.05) (Table 1).

### 3.2. Comparison of Anthropometric, Health, and Nutritional Characteristics by Nutritional Status

Significant differences in several anthropometric measures were observed between participants at risk of malnutrition and those with malnutrition. Median waist circumference was significantly higher in the at-risk group than in the malnutrition group among both males [93.00 (Q1–Q3: 86.00–102.67) cm vs. 85.00 (77.83–90.00) cm, *p* = 0.001] and females [95.00 (86.67–107.67) vs. 83.33 (80.00–91.33) cm, *p* = 0.035]. Waist circumference categories also differed significantly between the groups (*p* = 0.008), with abdominal obesity being more prevalent among participants at risk of malnutrition than among those with malnutrition (65.00% vs. 41.07%). In contrast, no significant difference was observed in waist-to-hip ratio categories (*p* = 0.415).

Participants with malnutrition had significantly lower calf circumference, mid-upper arm circumference, and BMI than those at risk of malnutrition. Median calf circumference was 25.50 (23.00–27.25) cm in the malnutrition group compared with 28.67 (26.00–32.75) cm in the at-risk group. Similarly, median mid-upper arm circumference was 23.33 (21.08–26.00) cm vs. 27.50 (25.08–31.00) cm, and median BMI was 16.36 (13.60–19.00) kg/m^2^ vs. 20.88 (17.81–26.14) kg/m^2^, respectively (all *p* < 0.001). BMI categories also differed significantly between the groups (*p* < 0.001). Underweight was substantially more prevalent among participants with malnutrition than among those at risk of malnutrition (71.43% vs. 32.50%), whereas obesity was more prevalent in the at-risk group (32.50% vs. 2.98%).

Functional status also differed significantly between the groups, with participants with malnutrition having a lower median Barthel ADL score than those at risk of malnutrition [0.00 (0.00–2.00) vs. 2.00 (0.00–3.00), *p* = 0.015]. However, the number of teeth and molar teeth did not differ significantly between the groups (*p* = 0.238 and *p* = 0.094, respectively).

Regarding macronutrient intake, no statistically significant differences were observed between participants at risk of malnutrition and those with malnutrition. Median carbohydrate intake was 384.74 (273.37–514.66) kcal in the at-risk group and 415.61 (309.44–518.84) kcal in the malnutrition group (*p* = 0.402). Corresponding median protein intake was 91.68 (68.78–134.95) vs. 106.29 (79.37–135.59) kcal (*p* = 0.236), while median fat intake was 130.80 (91.30–201.06) vs. 173.68 (111.99–241.78) kcal (*p* = 0.060). Total energy intake was also comparable between the groups [652.83 (455.30–818.38) vs. 677.64 (553.53–904.80) kcal, *p* = 0.220] (Table 2).

### 3.3. Factors Associated with Malnutrition Among Bedridden Older Adults

Multivariable binary logistic regression analysis was performed to identify factors independently associated with malnutrition among the 208 bedridden older adults. After simultaneous adjustment for all variables included in the model, sex and waist circumference were significantly associated with malnutrition.

Female participants had significantly higher odds of malnutrition than male participants (AOR = 2.302, 95% CI: 1.011–5.238, *p* = 0.047), indicating approximately 2.302-fold greater odds of being malnourished after adjustment for the other variables in the model.

Waist circumference was inversely associated with malnutrition (AOR = 0.948, 95% CI: 0.921–0.976, *p* < 0.001). Specifically, each 1 cm increase in waist circumference was associated with a 5.2% decrease in the odds of malnutrition [calculated as (1 − 0.948) × 100], after controlling for the other factors included in the model

In contrast, ability to eat was not significantly associated with malnutrition. Compared with the reference category of self-feeding and being fed by a caregiver, no significant associations were observed for self-feeding (AOR = 0.511, 95% CI: 0.181–1.444, *p* = 0.205), being fed by a caregiver (AOR = 1.185, 95% CI: 0.369–3.805, *p* = 0.776), or tube feeding (AOR = 3.370, 95% CI: 0.301–37.717, *p* = 0.324).

Barthel ADL score, number of molar teeth, carbohydrate intake, and protein intake were also not significantly associated with malnutrition (*p* > 0.05). Fat intake showed a positive association that approached, but did not reach, statistical significance (AOR = 1.005, 95% CI: 1.000–1.010, *p* = 0.059).

The overall logistic regression model was statistically significant (Omnibus χ^2^ = 36.572, df = 10, *p* < 0.001) and demonstrated acceptable goodness of fit according to the Hosmer–Lemeshow test (χ^2^ = 12.794, df = 8, *p* = 0.119). The model yielded a Nagelkerke R^2^ of 0.258. Although the overall classification accuracy was 83.7%, the model correctly classified 97.62% of participants with malnutrition and 25.00% of those at risk of malnutrition. Given the unequal distribution of the two nutritional-status groups, the overall classification accuracy was interpreted with caution. The AUC was 0.777 (95% CI: 0.694–0.860, *p* < 0.001), indicating acceptable discriminatory ability. The results of the multivariable logistic regression analysis are presented in Table 3.

## 4. Discussion

Among the 208 bedridden older adults included in the analysis, 40 (19.23%) were at risk of malnutrition and 168 (80.77%) were malnourished, indicating a substantial burden of compromised nutritional status in this population. The high proportion of malnutrition observed in this study highlights the nutritional vulnerability of bedridden older adults, who often experience functional limitations and dependence on others for daily care and food intake. This finding is consistent with a study of 315 Malawian older adults at hospital admission, which reported that 39.7% were at risk of malnutrition and 40.3% were malnourished, indicating a slightly higher proportion of malnourished participants than those at risk of malnutrition [29]. Regarding the demographic and household characteristics of bedridden older adults according to nutritional status, the majority of participants in the present study were classified as malnourished rather than at risk of malnutrition. This finding is consistent with the current situation, as the progressive aging of the population has been accompanied by a growing proportion of older adults who are either at risk of malnutrition or already malnourished [30,31]. This finding is also consistent with the present study, which was conducted in a community setting, and underscores the importance of continued nutritional monitoring and care among older adults, particularly those with mobility limitations and dependence on others for activities of daily living. This is especially important because poorer nutritional status has been associated with subsequent declines in functional independence [32]. In addition, the median total energy intake was relatively low at 671.22 kcal/day (IQR: 529.87–891.68). This finding may be related to limitations in activities of daily living (ADL), as the participants had a median ADL score of 1.00 (IQR: 0.00–2.00), indicating substantial functional limitations. This finding is consistent with previous research demonstrating that reduced physical function and frailty are associated with malnutrition [14]. However, functional dependence alone may not fully explain the low energy intake observed, and possible under-reporting associated with caregiver-reported dietary recalls should also be considered.

Female participants had significantly greater odds of malnutrition than male participants (AOR = 2.302, 95%CI: 1.011–5.238, *p* = 0.047), indicating that females had approximately 2.30 times higher odds of being malnourished after adjusting for the other variables in the model. This finding is consistent with a community-based cross-sectional study involving 739 randomly selected adults aged over 60 years in eastern Ethiopia [15], and to a similar study among older adults living in urban and rural areas in Southwest Ethiopia [17]. This association may be explained by the greater susceptibility of women to malnutrition (*p* = 0.004), possibly because women generally live longer and are more likely to outlive their partners [33]. In addition, the greater risk of malnutrition among female older adults may partly reflect lower physiological and muscle reserves. Following menopause, estrogen deficiency may contribute to reductions in skeletal muscle mass and strength through several mechanisms, including increased muscle cell apoptosis, impaired myosin phosphorylation, and altered satellite cell function [34]. These age- and sex-related changes may be particularly important among bedridden older adults, in whom prolonged physical inactivity can further accelerate muscle loss through reduced muscle protein synthesis [35]. Consistent with this mechanism, previous research found that 10 days of bed rest resulted in an approximately 30% reduction in the muscle protein fractional synthesis rate among healthy older adults, supporting the concept of anabolic resistance during bed rest [36]. Collectively, lower baseline muscle reserves combined with inactivity-related impairment in muscle protein synthesis may reduce nutritional and physiological reserves in bedridden older women, potentially increasing their vulnerability to malnutrition.

Waist circumference is a practical and reliable indicator of central adiposity and metabolic risk [37]. In the present study, waist circumference showed an inverse association with malnutrition (AOR = 0.948, 95% CI: 0.921–0.976, *p* < 0.001). After adjustment for other factors in the model, every 1 cm increase in waist circumference corresponded to a 5.2% lower odds of malnutrition [calculated as (1 − 0.948) × 100]. A similar finding was reported among 182 community-dwelling adults aged 65 years and older in Türkiye, where lower waist circumference was independently associated with a higher risk of malnutrition (OR: 1.089, 95% CI: 1.040–1.140, *p* < 0.001) [19]. Waist circumference represents visceral fat, and inflammation related to increased visceral fat may promote insulin resistance, which can subsequently reduce muscle mass and strength [38]. Low muscle mass is also incorporated into the most recent diagnostic criteria for malnutrition [39]. While waist circumference is generally related to abdominal obesity and metabolic disorders [40], in older adults, it may also indicate reduced lean muscle mass and has been associated with a higher risk of mortality [41]. The inverse association between waist circumference and malnutrition may simply reflect lower overall body mass and tissue stores among malnourished participants rather than a biologically protective effect of central adiposity.

In contrast, the ability to eat was not significantly associated with malnutrition. Using self-feeding and being fed by a caregiver as the reference category, no significant associations were found for self-feeding (AOR = 0.511, 95% CI: 0.181–1.444, *p* = 0.205), being fed by a caregiver (AOR = 1.185, 95% CI: 0.369–3.805, *p* = 0.776), or tube feeding (AOR = 3.370, 95% CI: 0.301–37.717, *p* = 0.324). Similarly, the Barthel ADL score was not significantly associated with malnutrition (*p* > 0.05), which may partly reflect the restricted ADL range (0–4) among the bedridden participants, limiting variability and statistical power to detect an association. However, the findings of the present study differ from previous evidence showing a positive association between nutritional status and functional status, with MNA^®^ scores positively correlated with Barthel Index scores [42].

The number of molar teeth was not significantly associated with malnutrition (*p* > 0.05), which differs from previous findings suggesting that oral factors may contribute to nutritional status in older adults. A study involving 986 adults aged ≥ 65 years recruited from 29 long-term care insurance facilities across 15 regions of Japan found that BMI < 20 kg/m^2^ was significantly associated with the number of molar occlusions (odds ratio: 0.52–0.70, 95% CI: 0.28–1.00) [22]. A lower number of occlusal contacts between functional teeth, including natural teeth, pontics, and dentures, has also been associated with poor nutritional intake among older adults requiring long-term care [22]. In our study, however, the number of molar teeth did not differ significantly between the at-risk-of-malnutrition and malnutrition groups and was therefore not significantly associated with malnutrition. The number of molar teeth alone may not adequately reflect chewing ability, as chewing function is closely related to both the number and distribution of remaining teeth [43].

Carbohydrate and protein intake were not significantly associated with malnutrition (*p* > 0.05). Fat intake showed a positive association that approached but did not reach statistical significance (AOR = 1.005, 95% CI: 1.000–1.010, *p* = 0.059). This may be related to the similar methods of food intake between the two groups, which may have resulted in comparable carbohydrate, protein, and fat intake. Previous evidence has shown that conditions that interfere with a person’s ability to eat can increase the risk of malnutrition [44].

## 5. Strengths and Limitations

This study focused on bedridden older adults, a vulnerable population with limited evidence regarding factors associated with malnutrition. A range of demographic, functional, anthropometric, oral health, and dietary factors were examined, and nutritional status was assessed using the MNA^®^.

Several limitations should be acknowledged. The cross-sectional analytical design does not allow temporal or causal relationships to be established, and the use of secondary data limited the analysis to variables available in the original dataset. Dietary intake data were obtained from caregiver-reported 24 h dietary recalls, which may be subject to recall error and under-reporting, particularly when caregivers were not present for all meals or were unable to accurately estimate the types and quantities of foods consumed. Consequently, the reported energy and nutrient intakes may not have fully reflected the participants’ actual dietary intake. In addition, potential selection bias cannot be excluded because the present study relied on participants recruited for the original study, whose sampling and eligibility procedures determined the population available for this secondary analysis. The number of molar teeth may not fully reflect chewing function or functional occlusion. The study was conducted in a single province, which may further limit the generalizability of the findings. Finally, only 40 participants were classified as being at risk of malnutrition. Given the number of parameters included in the multivariable logistic regression model, the relatively small number of participants in this group may have reduced the precision and stability of the coefficient estimates and increased the potential for model overfitting. Although the model demonstrated acceptable goodness of fit and discriminatory ability within the study sample, these findings do not exclude the possibility of overfitting. Therefore, the multivariable findings should be interpreted with caution, and further studies with larger and more balanced samples are needed to assess the stability and generalizability of the observed associations.

## 6. Future Directions

Future studies should employ prospective or longitudinal designs with larger and more diverse samples to better clarify the temporal relationships between associated factors and malnutrition among bedridden older adults. Given their substantial dependence on others for daily care and food intake, future research should place greater emphasis on caregiver-focused nutritional interventions. Such interventions should strengthen caregivers’ knowledge and skills in providing appropriate nutrition, preparing and modifying foods according to individual needs, assisting with feeding, and regularly monitoring nutritional status. Further studies should evaluate the effectiveness of these caregiver-based interventions in preventing malnutrition and improving nutritional outcomes among bedridden older adults.

## 7. Conclusions

This study provides evidence on factors associated with malnutrition among bedridden older adults in Nakhon Si Thammarat Province, Thailand. Among the demographic, functional, anthropometric, oral health, and dietary factors examined, sex and waist circumference were independently associated with malnutrition. Female bedridden older adults had higher odds of malnutrition than males, whereas a greater waist circumference was associated with lower odds of malnutrition. In contrast, ability to eat, Barthel ADL score, number of molar teeth, and carbohydrate, protein, and fat intake were not independently associated with malnutrition after adjustment for other factors.

These findings highlight the importance of routine nutritional assessment among bedridden older adults, with particular attention paid to anthropometric indicators and individuals who may be at greater nutritional risk. Waist circumference may provide useful complementary information for identifying nutritional vulnerability in this population; however, it should be considered alongside comprehensive nutritional assessment rather than used as a stand-alone indicator. Healthcare professionals, particularly nurses and primary care teams, should incorporate regular nutritional screening and anthropometric monitoring into the continuing care of bedridden older adults and use the findings to guide individualized nutritional care and timely intervention.

## Figures and Tables

**Table 1:** Comparison of demographic and health-related characteristics between risk of malnutrition and malnutrition groups (*n* = 208).

Personal Data	All Participants (*n* = 208)	Risk of Malnutrition (*n* = 40)	Malnutrition (*n* = 168)	χ^2^ or FET or U	*p*-Value
	*n* (%) or Median (Q1–Q3)	*n* (%) or Median (Q1–Q3)	*n* (%) or Median (Q1–Q3)		
Sex				χ^2^ = 2.57	0.143
Male	76 (36.54)	19 (47.50)	57 (33.93)		
Female	132 (63.46)	21 (52.50)	111 (66.07)		
Age (Years)	82.00 (73.00–89.00)	81.85 (74.25–88.75)	82.00 (73.00–89.08)	U = 3230.50	0.705
Religion				FET = 0.50	0.581
Buddhism	186 (89.42)	37 (92.50)	149 (88.69)		
Islam	22 (10.58)	3 (7.50)	19 (11.31)		
Marital status				χ^2^ = 0.10	0.963
Single	14 (6.73)	3 (7.50)	11 (6.55)		
Married	86 (41.35)	17 (42.50)	69 (41.07)		
Divorced/Widowed/Separated	108 (51.92)	20 (50.00)	88 (52.38)		
Duration of bedridden status (months)	30.00 (12.00–50.25)	36.00 (14.75–60.00)	29.00 (12.00–48.00)	U = 3003.50	0.297
Ability to eat				χ^2^ = 9.85	0.020
Self-feeding	49 (23.56)	16 (40.00)	33 (19.64)		
Fed by caregiver	91 (43.75)	14 (35.00)	77 (45.83)		
Tube feeding	23 (11.06)	1 (2.50)	22 (13.10)		
Self-feeding and Fed by caregiver	45 (21.63)	9 (22.50)	36 (21.43)		
Number of family members	3.00 (3.00–5.00)	4.00 (3.00–5.00)	3.00 (3.00–5.00)	U = 3281.50	0.814
Primary caregiver				FET = 3.86	0.246
Spouse	48 (23.08)	11 (27.50)	37 (22.02)		
Child	124 (59.62)	19 (47.50)	105 (62.50)		
Relative	32 (15.38)	9 (22.50)	23 (13.69)		
Paid caregiver	4 (1.92)	1 (2.50)	3 (1.79)		
Monthly household income	10,000.00 (5000.00–19,500.00)	10,000.00 (5000.00–20,000.00)	10,000.00 (5000.00–17,850.00)	U = 3327.50	0.924
Income sufficiency				χ^2^ = 0.59	0.714
More than sufficient	7 (3.37)	2 (5.00)	5 (2.98)		
Sufficient	68 (32.69)	14 (35.00)	54 (32.14)		
Insufficient	133 (63.94)	24 (60.00)	109 (64.88)		

χ^2^ = Chi-square test; FET = Fisher’s exact test; U = Mann–Whitney U test.

**Table 2:** Anthropometric characteristics, health status, and macronutrient intake.

Health Status	All Participants (*n* = 208)	Risk of Malnutrition (*n* = 40)	Malnutrition (*n* = 168)	χ^2^ or FET or U	*p*-Value
	*n* (%) or Median (Q1–Q3)	*n* (%) or Median (Q1–Q3)	*n* (%) or Median (Q1–Q3)		
Waist circumference (cm)					
Male (*n* = 76)	85.83 (80.25–93.00)	93.00 (86.00–102.67)	85.00 (77.83–90.00)	U = 267.00	0.001
Female (*n* = 132)	84.83 (80.00–95.00)	95.00 (86.67–107.67)	83.33 (80.00–91.33)	U = 826.50	0.035
Waist circumference categories				χ^2^ = 7.46	0.008
Normal (Male < 90, Female < 80)	113 (54.33)	14 (35.00)	99 (58.93)		
Abdominal obesity (Male ≥ 90, Female ≥ 80)	95 (45.67)	26 (65.00)	69 (41.07)		
Waist/Hip ratio				χ^2^ = 1.77	0.415
Low risk (Male < 0.90, Female < 0.80)	35 (16.83)	8 (20.00)	27 (16.07)		
Moderate risk (Male = 0.90–0.99, Female = 0.80–0.84)	40 (19.23)	10 (25.00)	30 (17.86)		
High risk (Male ≥ 1.00, Female ≥ 0.85)	133 (63.94)	22 (55.00)	111 (66.07)		
Calf circumference (cm)	26.00 (23.00–28.00)	28.67 (26.00–32.75)	25.50 (23.00–27.25)	U = 1722.00	<0.001
Mid-upper arm circumference (cm)	24.00 (22.00–27.33)	27.50 (25.08–31.00)	23.33 (21.08–26.00)	U = 1493.00	<0.001
BMI (kg/m^2^)	16.79 (13.95–19.84)	20.88 (17.81–26.14)	16.36 (13.60–19.00)	U = 1564.00	<0.001
BMI categories				FET = 34.30	<0.001
Underweight (<18.50)	133 (63.94)	13 (32.50)	120 (71.43)		
Normal (18.50–22.99)	51 (24.52)	12 (30.00)	39 (23.21)		
Overweight (23–24.99)	6 (2.88)	2 (5.00)	4 (2.38)		
Obesity (≥25.00)	18 (8.66)	13 (32.50)	5 (2.98)		
Barthel ADL score	1.00 (0.00–2.00)	2.00 (0.00–3.00)	0.00 (0.00–2.00)	U = 2580.50	0.015
Number of teeth	6.00 (6.00–18.00)	9.00 (0.00–23.75)	5.50 (0.00–17.75)	U = 2968.50	0.238
Number of molar teeth	2.00 (0.00–6.00)	3.00 (0.00–8.00)	0.00 (0.00–5.50)	U = 2769.50	0.094
Carbohydrate intake (kcal)	408.30 (297.76–518.84)	384.74 (273.37–514.66)	415.61 (309.44–518.84)	U = 3073.00	0.402
Protein intake (kcal)	105.11 (74.56–135.18)	91.68 (68.78–134.95)	106.29 (79.37–135.59)	U = 2955.00	0.236
Fat intake (kcal)	167.44 (104.70–237.60)	130.80 (91.30–201.06)	173.68 (111.99–241.78)	U = 2717.00	0.060
Total energy (kcal)	671.22 (529.87–891.68)	652.83 (455.30–818.38)	677.64 (553.53–904.80)	U = 2940.00	0.220

χ^2^ = Chi-square test; FET = Fisher’s exact test; U = Mann-Whitney U test.

**Table 3:** Multivariate logistic regression analysis of factors associated with malnutrition among bedridden older adults (*n* = 208).

Factors	B	SE	Wald	AOR (95%CI)	*p*-Value
Sex					
Male (Reference)					
Female	0.834	0.420	3.947	2.302 (1.011–5.238)	0.047
Ability to eat					
Self-feeding and fed by caregiver (Reference)					
Self-feeding	−0.672	0.530	1.606	0.511 (0.181–1.444)	0.205
Fed by caregiver	0.170	0.595	0.081	1.185 (0.369–3.805)	0.776
Tube feeding	1.215	1.232	0.972	3.370 (0.301–37.717)	0.324
Waist circumference (cm)	−0.053	0.015	12.820	0.948 (0.921–0.976)	<0.001
Barthel ADL score	−0.115	0.173	0.442	0.892 (0.636–1.251)	0.506
Number of molar teeth	−0.041	0.041	0.967	0.960 (0.885–1.041)	0.326
Carbohydrate intake (kcal)	−0.002	0.001	1.989	0.998 (0.996–1.001)	0.158
Protein intake (kcal)	0.001	0.005	0.022	1.001 (0.991–1.010)	0.882
Fat intake (kcal)	0.005	0.003	3.571	1.005 (1.000–1.010)	0.059

## Data Availability

The data supporting the findings of this study are available from the corresponding author upon reasonable request. The data are not publicly available due to privacy and ethical considerations.
